# Red blood cell differentiation using canine-induced pluripotent stem cells

**DOI:** 10.1093/stcltm/szag058

**Published:** 2026-07-20

**Authors:** Kazuto Kimura, Masaya Tsukamoto, Kohei Shishida, Hiroko Sugisaki, Jun Katahira, Miyuu Tanaka, Mitsuru Kuwamura, Amir Kol, Mika Okada, Minoru Iijima, Mahito Nakanishi, Kikuya Sugiura, Shingo Hatoya

**Affiliations:** Laboratory of Cell Pathobiology, Department of Advanced Pathobiology, Graduate School of Veterinary Science, Osaka Metropolitan University, Izumisano, 598-8531, Japan; Department of Pathology, Microbiology and Immunology, School of Veterinary Medicine, University of California, Davis, Davis, CA 95616, United States; Laboratory of Cell Pathobiology, Department of Advanced Pathobiology, Graduate School of Veterinary Science, Osaka Metropolitan University, Izumisano, 598-8531, Japan; Laboratory of Cell Pathobiology, Department of Advanced Pathobiology, Graduate School of Veterinary Science, Osaka Metropolitan University, Izumisano, 598-8531, Japan; Laboratory of Cell Pathobiology, Department of Advanced Pathobiology, Graduate School of Veterinary Science, Osaka Metropolitan University, Izumisano, 598-8531, Japan; Laboratory of Cellular Molecular Biology, Department of Integrated Functional Biosciences, Graduate School of Veterinary Science, Osaka Metropolitan University, Izumisano, 598-8531, Japan; Laboratory of Veterinary Pathology, Department of Integrated Structural Biosciences, Graduate School of Veterinary Science, Osaka Metropolitan University, Izumisano, 598-8531, Japan; Laboratory of Veterinary Pathology, Department of Integrated Structural Biosciences, Graduate School of Veterinary Science, Osaka Metropolitan University, Izumisano, 598-8531, Japan; Department of Pathology, Microbiology and Immunology, School of Veterinary Medicine, University of California, Davis, Davis, CA 95616, United States; TOKIWA-Bio Inc., Tsukuba, 305-0047, Japan; TOKIWA-Bio Inc., Tsukuba, 305-0047, Japan; TOKIWA-Bio Inc., Tsukuba, 305-0047, Japan; Laboratory of Cell Pathobiology, Department of Advanced Pathobiology, Graduate School of Veterinary Science, Osaka Metropolitan University, Izumisano, 598-8531, Japan; Laboratory of Cell Pathobiology, Department of Advanced Pathobiology, Graduate School of Veterinary Science, Osaka Metropolitan University, Izumisano, 598-8531, Japan

**Keywords:** red blood cells, differentiation, canine, induced pluripotent stem cells, genome editing

## Abstract

**Background:**

Red blood cell (RBC) transfusions are essential for treating various medical conditions, but global demand is difficult to meet due to a dwindling donor pool and compatibility issues. Pluripotent stem cells (PSCs) offer a promising alternative of blood dependent on volunteer donors for RBC production, and dogs serve as an excellent model for translational research due to their physiological and genetic similarities to humans.

**Methods:**

Canine induced pluripotent stem cells (ciPSCs) were differentiated toward hematopoietic and erythroid lineages. Differentiated cells were evaluated for hematopoietic marker expression, hemoglobinization, colony-forming capacity, enucleation, and hemoglobin gene expression. Glycophorin A (GYPA)-enhanced green fluorescent protein (EGFP) reporter ciPSC lines were generated using clustered regularly interspaced short palindromic repeats (CRISPR)-Cas9–mediated genome editing to visualize GYPA expression during differentiation.

**Results:**

This study introduces a protocol for RBC differentiation using ciPSCs. We achieved generation of hemoglobinized RBCs, progressing through polychromatic and orthochromatic erythroblast–like stages. CiPSC–derived hematopoietic cells/RBCs were confirmed to have immature characteristics as determined by limited colony-forming capacities, low enucleation, and embryonic and fetal hemoglobin gene expression. Additionally, we created GYPA-EGFP reporter ciPSC lines using CRISPR-Cas9–mediated genome editing, enabling real-time visualization of GYPA expression. This innovation confirmed GYPA as a viable surface marker for ciPSC-derived RBCs.

**Conclusion:**

Our findings mark an initial step toward establishing a canine PSC–based erythroid differentiation system, providing a foundation for future improvements and exploration of applications for canine PSC–derived RBCs.

## Graphical abstract

**Graphical Abstract:**
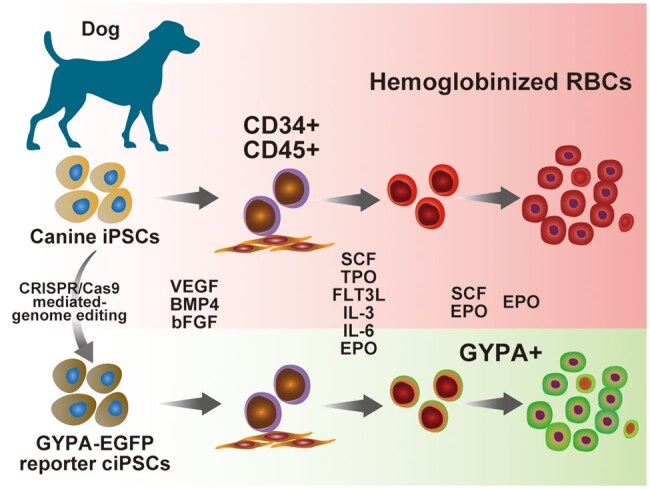

Significance statement This study explores a novel approach to red blood cell (RBC) production using canine-induced pluripotent stem cells (ciPSCs) as a preliminary step toward addressing challenges in veterinary blood supply. We developed a differentiation protocol and created GYPA-EGFP reporter ciPSC lines to facilitate monitoring of erythroid induction. Although further optimization and functional validation are required, this research provides a foundation for future efforts to improve ciPSC-derived RBC production and may ultimately contribute to advance in veterinary medicine and comparative research.

## Introduction

Red blood cell (RBC) transfusion is a vital therapy for various conditions, including significant bleeding during surgeries, severe anemia, trauma from accidents, and genetic blood disorders such as sickle cell disease and β-thalassemia. However, meeting the global demand for blood transfusions is becoming increasingly challenging due to a decline in blood donors caused by an aging population, the need for compatibility with RBC antigens like ABO and Rh, and the risk of severe transfusion-transmissible infections such as HIV and hepatitis B and C from unscreened donations.^1^^,^^2^ Instead of relying on blood donations, pluripotent stem cells (PSCs) offer a promising alternative. Pluripotent stem cells have an almost infinite capacity for self-renewal and can differentiate into various cell types, making them an attractive resource for RBC production. Recent advances in *ex vivo* RBC generation technologies and cell engineering have made this approach more feasible. Moreover, O-negative and Rh-negative RBCs are considered “universal” donors, suitable for transfusion to recipients of almost all blood types.^3^ Consequently, generating induced pluripotent stem cells (iPSCs) from universal donors could provide a stable supply of universal RBCs for nearly all recipients.

Dogs are widely recognized as companion animals, which increases the demand for advanced veterinary medicine. Furthermore, dogs offer several advantages over classical rodent models, making them invaluable for translational research in human medicine.^4^ Unlike rodent models, dogs have a longer lifespan and naturally develop many counterparts of human conditions, such as cancer, heart disease, and diabetes. Their physiology, genetics, and living environments are also quite similar to those of humans.^5^ Therefore, clinical trials in veterinary medicine can provide unique and reliable data on the long-term effects and safety of treatments, whereas preclinical studies in mice often fail to predict human clinical trial outcomes.^6^^,^^7^ In canines, DEA1.1-negative RBCs, like human universal RBCs, can be transfused to many recipients.^8^ Thus, using canine-induced pluripotent stem cell (ciPSC)–derived DEA1.1-negative RBCs could serve as an attractive model for universal RBC transfusion in human medicine.

Numerous methods for differentiating RBCs from human PSCs (hPSCs) have been reported, typically by mimicking mammalian hematopoiesis *in vitro*. In mice, erythropoiesis occurs in 3 distinct waves.^9^ The first wave, from embryonic day 7 (E7) to E9, involves the emergence of large, nucleated primitive erythrocytes expressing embryonic hemoglobin from hemangioblasts, endothelial-like cells in the yolk sac.^10^^,^^11^ The second wave starts at E8.25, with erythroid-myeloid progenitors arising from hemogenic endothelium (HE) in the yolk sac’s blood islands and differentiating into definitive erythrocytes.^12–14^ The third wave begins at E10.5, with hematopoietic stem/progenitor cells (HSPCs) emerging in the aorta-gonad-mesonephros region and other embryonic arterial vessels, the yolk sac, and the placenta, from which definitive erythrocytes are subsequently derived.^13^^,^^15^^,^^16^ The RBC differentiation process using hPSCs mimics these developmental stages *in vitro*, leading to the simultaneous induction of erythrocytes from the 3 waves, albeit with some variation in proportions.^17–21^ For applications in transfusion, it is necessary to produce enucleated definitive RBCs that express adult-type hemoglobin from iPSCs; however, a robust method for achieving this is still unclear. Additionally, in canines, RBC differentiation methods using ciPSCs have not yet been elucidated.

Glycophorin A (GYPA, also known as CD235a) is a major sialoglycoprotein of the human erythrocyte membrane, involved in cell-cell interactions, signaling, and maintaining red cell membrane stability.^22^ Therefore, GYPA is a useful specific cell surface marker for human erythrocytes even after enucleation. Furthermore, it is reported that during the early stages of differentiation, GYPA is expressed exclusively in primitive, but not in definitive, hematopoietic progenitor cells derived from hPSCs, indicating that GYPA serves as a marker to distinguish between these two lineages.^23^ However, anti-canine GYPA antibodies are not commercially available, and some studies reported that the anti-human GYPA antibodies did not cross to canine GYPA,^24^ complicating the quantification of ciPSC-derived RBCs. In contrast, recent advancements in genome-editing technology, such as the clustered regularly interspaced short palindromic repeats (CRISPR)-Cas9 system,^25^ have enabled the knock-in of reporter genes like enhanced green fluorescent protein (EGFP) into targeted gene loci, facilitating the generation of reporter cell lines.^26–28^ These reporter PSC lines allow for real-time visualization of gene expression in cells during differentiation.

In this study, we describe a protocol for the differentiation of RBCs from ciPSCs and examine their characteristics. Furthermore, we demonstrate the successful generation of reporter ciPSCs using CRISPR-Cas9–mediated genome editing. These reporter ciPSCs allowed for the real-time visualization of GYPA expression in ciPSC-derived RBCs, facilitating the quantification of RBCs without the need for antibody-based detection.

## Methods

### Material availability

There are restrictions to the availability of ciPSC lines and GYPA-EGFP reporter ciPSC lines due to the lack of an external centralized repository for its distribution and our need to maintain the stock. We are glad to share them with reasonable compensation by requestor for its processing and shipping.

### Animals and ethics statement

This study was approved by the Institutional Animal Experiment Committee of Osaka Metropolitan University (permission numbers: 22-73, 22-74, 23-53, 23-56). We performed this study according to the Animal Experimentation Regulations of Osaka Metropolitan University.

### Culture medium composition

The Stemline II-based medium was composed of Stemline II (Sigma-Aldrich), 1% Chemically Defined Lipid Concentrate (Thermo Fisher Scientific), 1% insulin-transferrin-selenium-ethanolamine supplement (ITS supplement; Sigma-Aldrich), 100 U/mL penicillin, and 100 µg/mL streptomycin (Nacalai Tesque). The Iscove’s Modified Dulbecco’s Medium (IMDM)–based medium was composed of IMDM (Sigma-Aldrich) containing 10% fetal bovine serum (FBS; Sigma-Aldrich), 1% GlutaMAX (Thermo Fisher Scientific), 1% Chemically Defined Lipid Concentrate, 10 µg/mL insulin (Sigma-Aldrich), 200 µg/mL holo-transferrin (Fujifilm Wako Pure Chemical Corporation), 100 U/mL penicillin, and 100 µg/mL streptomycin.

### ciPSC culture

Canine-induced pluripotent stem cell lines were generated at our laboratory from canine peripheral blood mononuclear cells (PBMCs) (OPUiD04-B, OPUiD05-A^29^ and OPUiD01-CPB [generated in this study]) and dermal fibroblast [DF] (OPUiD05-FA-1).^30^ Canine-induced pluripotent stem cells were maintained on iMatrix-511 silk (0.25 µg/cm^2^, Nippi, Inc.)–coated dishes in StemFit AK02N medium (StemFit; Ajinomoto) at 5% CO_2_ and 37 °C. The medium was changed every day. Canine-induced pluripotent stem cells were passaged mechanically using a glass Pasteur pipette with a split ratio of 1:5 to 1:20 every 3-5 days or enzymatically using 0.5× TrypLE Select (1× TrypLE Select [Thermo Fisher Scientific] diluted 1:1 with 0.5 mM ethylenediaminetetraacetic acid [Nacalai Tesque]/D-PBS (–)) at a density of 1-3 × 10^3^/cm^2^ every 3-5 days. The cells were cultured in StemFit supplemented with 10 µM Y-27632 (Nacalai Tesque) for only 24 h after passaging. For cryopreservation, ciPSCs were dissociated with 0.5× TrypLE Select and stored at −80 °C or −196°C using STEM-CELLBANKER (Nippon Zenyaku Kogyo). Canine-induced pluripotent stem cells at passages 15-40 were used for further experiments.

### Differentiation of ciPSCs into RBCs

For differentiation, on day −1, dissociated ciPSCs by 0.5× TrypLE Select were plated into Nunclon Sphera 96-Well U-Shaped-Bottom Microplate (Thermo Fisher Scientific) in StemFit supplemented with 10 µM Y-27632 at 4 × 10^4^ cells/well. On day 0, formed embryoid bodies (EBs) were plated onto gelatin-coated tissue culture 6-well plates at 6 EBs/well and cultured for 6 days in the Stemline II–based medium supplemented with 50 ng/mL human basic fibroblast growth factor 2 (Peprotech), 20 ng/mL human bone Morphogenetic protein 4 (Miltenyi Biotec), and 30 ng/mL human vascular endothelium growth factor (Thermo Fisher Scientific). On day 6, medium was changed into Stemline II–based medium supplemented with 50 ng/mL canine stem cell factor (cSCF), 50 ng/mL human thrombopoietin (Peprotech), 50 ng/mL human Fms–like tyrosine kinase 3 ligand (Peprotech), 5 ng/mL canine interleukin 3 (Novus), 5 ng/mL canine interleukin 6 (Prospec), and 3 U/mL canine erythropoietin (cEPO). On day 12, the differentiated cells in plates were dissociated by 0.5× TrypLE Select and replated onto gelatin-coated tissue culture 6-well plates in the IMDM-based medium supplemented with 50 ng/mL cSCF, and 3 U/mL cEPO. On day 18, medium was changed to IMDM-based medium supplemented with 3 U/mL cEPO and cultured until day 24. The medium was changed every 3 days through differentiation.

### Cytospins and staining

Briefly, 1 × 10^5^ cells were centrifuged onto glass microscope slides using Cytospin 4 (Thermo Fisher Scientific). The slides were air-dried and Wright/Giemsa stained. The cytospin slides were incubated for 5 min in Wright solution followed by 15 min in phosphate buffer and with subsequent staining in Giemsa solution for 30 min.

### Flow cytometry

For flow cytometry (FCM), the cells were dissociated using 0.5× TrypLE Select. Cell pellets were resuspended in FCM buffer composed of PBS (–) containing 2% FBS and 1 mg/mL sodium azide (Fujifilm Wako Pure Chemical Corporation) and labeled with SSEA-1, CD34-PE, CD45-FITC, and 10 µM DRAQ5 (BioStatus Ltd.) on ice for 30 min. For SSEA-1 staining, after washing in FCM buffer, cells were labeled with Cy3 goat anti-mouse IgM as secondary antibody (Sigma-Aldrich) on ice for 15 min. Negative control cells were incubated with the isotype control. The cells were washed in FCM buffer and analyzed using FCM (CytoFLEX; Beckman Coulter).

### Colony-forming assay

About 50 000 ciPSC-derived hematopoietic cells on differentiation day 7 were cultured in 1.2 mL cytokine-containing MethoCult M3434 medium (StemCell Technologies) supplemented with 50 ng/mL canine granulocyte-macrophage colony-stimulating factor (GM-CSF) (R&D Systems) at 37 °C. After 14 days, the hematopoietic colonies were scored for colony-forming units according to cellular morphology.

### Reverse transcription polymerase chain reaction and quantitative reverse transcription polymerase chain reaction

Total RNA was isolated using a FastGene RNA Premium Kit (Nippon Genetics) and reverse transcribed into complementary DNA using random primers and ReverTra Ace (Toyobo). Polymerase chain reaction (PCR) was performed using a Blend Taq Plus (Toyobo). Polymerase chain reaction products were resolved on a 2% agarose gel stained with ethidium bromide and observed using an ultraviolet transilluminator (AE-9020; ATTO).

To quantify mRNA expression levels, PCR was performed using Taq Pro Universal SYBR qPCR Master Mix (Nanjing Vazyme Biotech) and a Step One Plus Real-Time PCR System (Thermo Fisher Scientific). *β-ACTIN* was used as a normalization control gene, and relative gene expression levels were calculated via the ΔΔCt method. All primers are listed in Table S1.

### Immunocytochemistry

The cells were fixed in 4% paraformaldehyde, permeabilized with 0.1% Tween 20 or Triton X-100 in PBS (−), and blocked with 10% bovine serum albumin (Nacalai Tesque). The cells were then incubated with primary antibodies at 4 °C overnight. Negative control cells were incubated in PBS (−) without primary antibodies. The next day, the cells were washed, incubated with appropriate secondary antibodies at 25 °C for 1 h, and mounted using ProLong Gold Antifade Reagent with 4′,6-diamidino-2-phenylindole (Thermo Fisher Scientific) to label DNA. Immunolabeled cells were observed using a confocal laser-scanning microscope (FV3000; Olympus). All antibodies are listed in Table S2.

### sgRNA design and Cas9 vector assembly

Cas9 target sites were identified using the online CRISPR design tool (crispr.mit.edu). Briefly, 200-bp DNA sequences of the canine GYPA gene flanking the stop codon (100 bp before and after the stop codon) were used for designing the sgRNAs. Later on, 1 pair of sgRNAs was selected and then for each target site, a specific Cas9 vector was made. Briefly, the pX330-U6-Chimeric_BB-CBh-hSpCas9 (pX330; #42230 from Addgene) was digested using BbsI and a pair of annealed oligos was cloned into the guide RNA locus. Finally, vectors were sequenced to ensure the presence of the right sequence. sgRNA sequence is shown in Table S3.

### Targeting vector design and construct

The targeting vector, pNT1.1 vector,^31^ was provided by Dr Masato Ikawa, Department of Experimental Genome Research, Research Institute for Microbial Diseases, Osaka University. For construction of the targeting vector, the homology arms along with the target region and the EGFP reporter were cloned into pNT1.1 vector through a gateway recombination after digestion using XhoI and PmeI.

### Generation of the GYPA reporter ciPSC clones and selection

A fully characterized integration-free ciPSC line, OPUiD05-A, was used for generation of canine reporter clones. The ciPSCs were expanded on iMatrix-511 silk in StemFit for a few days and after harvesting, 8 × 10^5^ cells were electroporated using 5 µg of pX330 vector plus 5 µg of pNT1.1 vector with the program B-016 in Nucleofector2b (LONZA). After the electroporation, cells were expanded for a few days and selection was done by adding positive selection 200 µg/mL G-418 solution (Fujifilm Wako Pure Chemical Corporation) for approximately 7 days. After the selection, ciPSC colonies were mechanically picked up for expansion and PCR screening.

### PCR screening of reporter clones

Genomic DNA was isolated from each clone by treatment with proteinase K (Fujifilm Wako Pure Chemical Corporation) or the PureLink Genomic DNA Mini Kit (Thermo Fisher Scientific). Polymerase chain reaction was performed using the Expand High Fidelity PCR System Blend Taq Plus (Sigma-Aldrich) followed by the manufacturer’s instrument. Polymerase chain reaction products were resolved on a 1% agarose gel stained with ethidium bromide and observed using an ultraviolet transilluminator. All primers used in this experiment are listed in Table S3.

### Digital PCR

Genomic copy number of the EGFP knock-in cassette was quantified using the QIAcuity One 5-plex digital PCR (dPCR) system with 8.5k nanoplates (Qiagen). The assay was multiplexed with a previously reported single-copy autosomal reference target within *MC1R*.^32^ Reactions were prepared using the QIAcuity Probe PCR Kit (Qiagen) according to the manufacturer’s instructions, with primers and probes used at final concentrations of 0.8 and 0.4 µM, respectively. Genomic DNA was digested directly in the QIAcuity reaction mixture with HindIII-HF restriction enzyme (New England Biolabs, Inc.) for 10 min at room temperature prior to loading. The reaction mixtures were then loaded onto 8.5k nanoplates and subjected to dPCR under the following cycling conditions: 95 °C for 2 min, followed by 40 cycles of 94 °C for 30 s and 59 °C for 1 min. Following amplification, nanoplates were imaged in the green, yellow, red, and crimson channels. Copy number variation data were analyzed using the QIAcuity Software Suite (v2.2.0.26; Qiagen), with *MC1R* set as the reference target and assumed to be present at 2 copies per genome. All samples were analyzed in technical duplicate, and 95% CIs were calculated automatically by the software. Primer and probe sequences used in this study are listed in Table S4.

### Western blot

The cells derived from GYPA-reporter ciPSCs at days 6, 12, 18, and 24 of differentiation were mixed with 1× sample buffer composed of 100 mM Tris-HCl, 1.6% sodium dodecyl sulfate (SDS), 8% glycerol, 5% 2-mercaptoethenol, and 0.08% bromophenol blue (BPB) and lysed using a sonicator. The lysates were subjected to SDS-PAGE using Extra PAGE One Precast Gel (Nacalai Tesque). Following SDS-PAGE, the proteins were transferred onto a polyvinylidene fluoride (PVDF) membrane at 15 V for 90 min. The membrane was then blocked overnight at 4 °C in Tris-buffered saline with 0.1% Tween 20 (TBST) buffer composed of 20 mM Tris-HCl (pH 7.4), 150 mM NaCl, and 0.05% Tween 20 containing 5% skim milk. After blocking, the membrane was incubated with the rabbit anti–GFP polyclonal antibody (1:2000, A-6455, Thermo Fisher Scientific) diluted in TBST containing 3% skim milk for 1 hr at room temperature. The membrane was washed 3 times for 5 min each with TBST, followed by incubation with goat anti–rabbit HRP-conjugated antibody (1:3000, 1706515, Bio-Rad Laboratories) diluted in TBST containing 3% skim milk for 1 hr at room temperature. After incubation with secondary antibodies, the membrane was washed 3 times for 5 min each with TBST. The signal was detected using Chemi-Lumi One (Nacalai Tesque) as the substrate, and chemiluminescence was imaged using the iBright CL1500 Imaging System (Thermo Fisher Scientific).

### Statistical analysis

Data are expressed as the mean ± SD. Statistical significance was determined using the Tukey-Kramer multiple comparison procedure and Statcel software (OMS Ltd.).

## Results

### RBC differentiation protocol using ciPSCs

**Figure 1.**
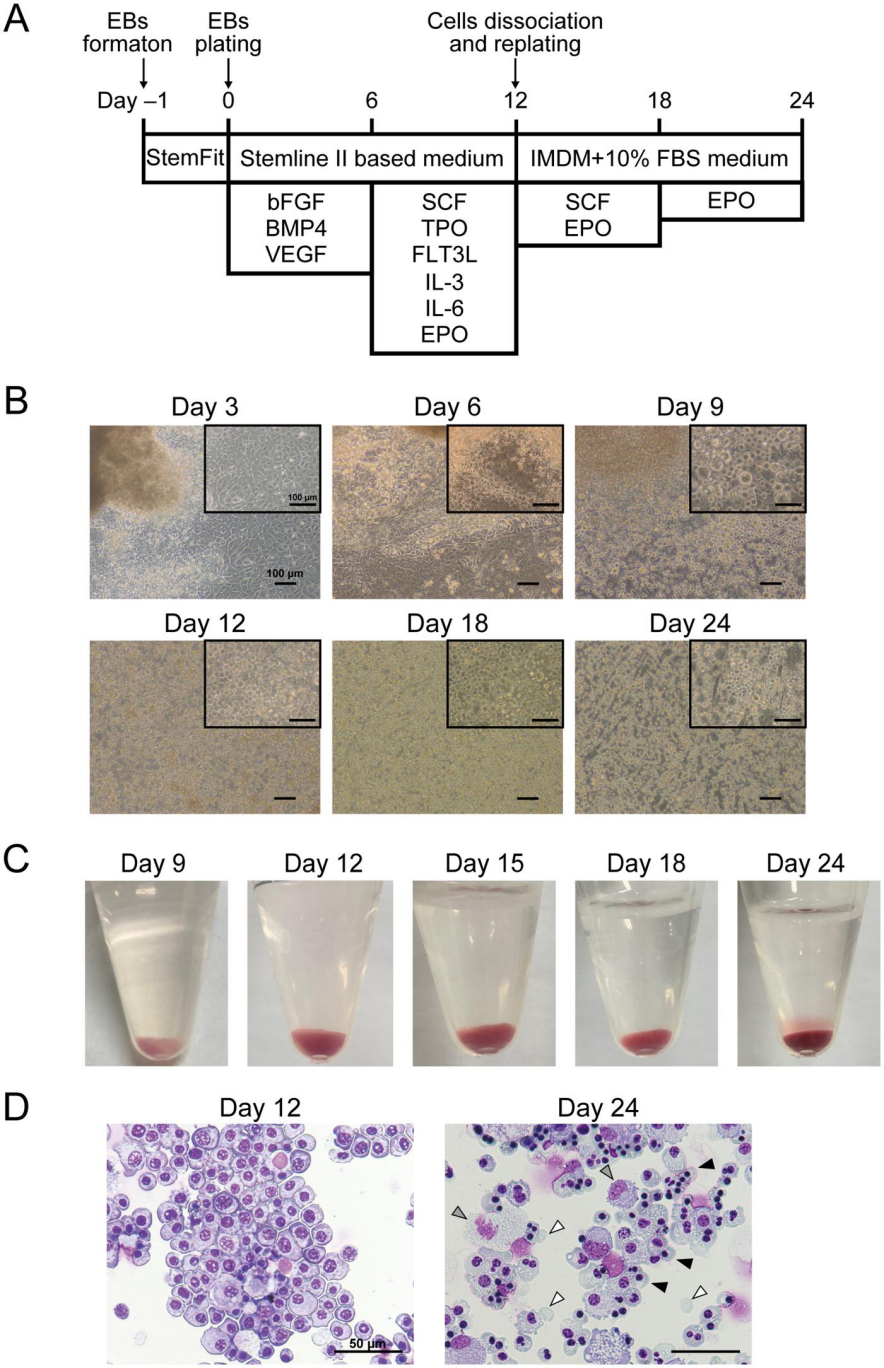
RBC differentiation from ciPSCs. (A) Scheme of RBC differentiation protocol. (B) Morphology of differentiated cells at each time point. Scale bar = 100 µm. (C) Morphology of differentiated cell pellets at each time point. (D) Morphology of the differentiated cells by Wright/Giemsa staining on days 12 and 24. EBs: embryoid bodies, IMDM: Iscove’s Modified Dulbecco’s Medium, FBS: fetal bovine serum, bFGF: basic fibroblast growth factor, BMP4: bone marrow protein 4, VEGF: vascular endothelium growth factor, SCF: stem cell factor, TPO: thrombopoietin, FLT3L: Fms–like tyrosine kinase 3 ligand, IL-3: interleukin 3, IL-6: interleukin 6, EPO: erythropoietin. Black arrows: orthochromatic erythroblast–like cells, white arrows: enucleated erythrocytes, gray arrows macrophage-like cells. Scale bar = 50 µm.

We first tried to differentiate ciPSCs into RBCs using the HSPC/RBC differentiation protocol for hPSCs.^33^ Although the spherical cells were obtained in some experiments, it was not reproducible (data not shown). To improve the reproducibility of the protocol, we combined another efficient HE differentiation protocol for hPSCs,^34^ in which the spheroids are formed from hPSCs and are spontaneously re-flatten on the plates, with the former protocol.^33^ The scheme of our protocol to differentiate ciPSCs into RBCs is shown in Figure 1A. We formed EBs by seeding ciPSCs onto 96-well U-shaped-bottom plates and then reseeded them onto cell culture plates on the next day. One ciPSC line, OPUiD05-A, which has been confirmed to have the teratoma formation ability,^29^ was utilized in this experiment. Canine-induced pluripotent stem cell–derived EBs were attached to the bottom of the cell culture plates and differentiated into the endothelial-like cells around day 3 (Figure 1B). On day 6, the spherical cells began to be released from the endothelial-like cells (Figure 1B). The number of them increased until day 18 and then decreased as the culture continued until day 24 (Figure 1B). These morphological changes were observed in almost all experiments, suggesting that this protocol would be reproducible. Following induction of RBC differentiation, the cell pellets showed gross evidence of hemoglobinization (Figure 1C). To evaluate the morphological characteristics of the differentiated cells at some time points, we performed Wright/Giemsa staining. On day 12, many cells exhibited a morphology of polychromatic erythroblast–like cells (Figure 1D). By the end of the differentiation process on day 24, numerous cells displayed the morphology of orthochromatic erythroblast–like cells, along with a few enucleated erythrocytes and some macrophage-like cells (Figure 1D).

### Marker expression and hematopoiesis potentials of the differentiated cells in early differentiation stage

**Figure 2.**
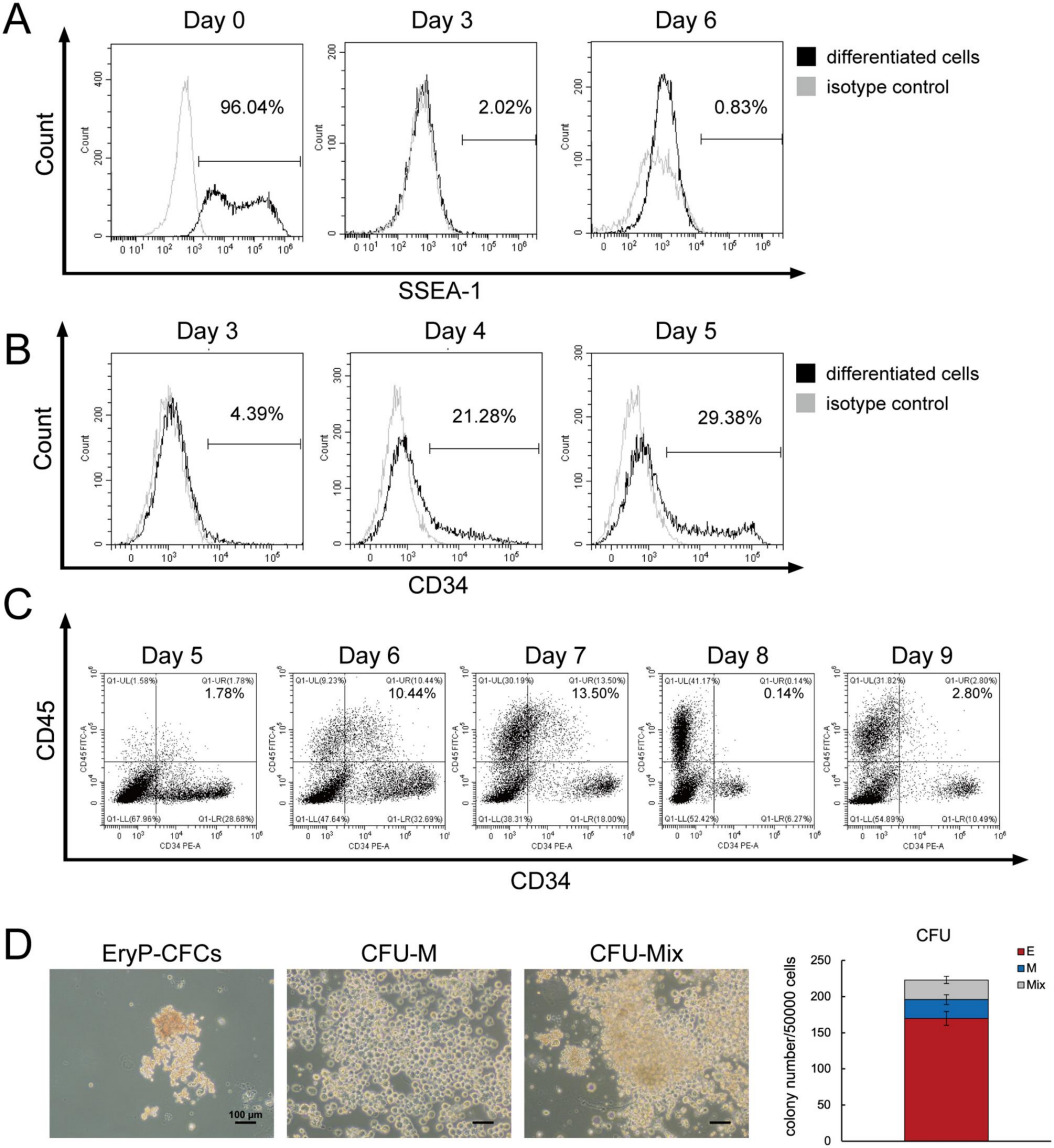
Marker expressions and hematopoiesis potentials of differentiated cells. (A) Flow cytometric analysis of the differentiated cells for SSEA-1 at each time point. Black line: differentiated cells. Gray line: isotype control. (B) Flow cytometric analysis of the differentiated cells for CD34 from days 3 to 5. Black line: differentiated cells. Gray line: isotype control. (C) Flow cytometric analysis of the differentiated cells for CD34 and CD45 from days 5 to 9. (D) Colony-forming assay of the differentiated cells on day 7. Left panels show the representative morphology of each colony. Right panel shows the number of each colony. Data are shown as the mean ± SD (*n* = 3). EryP-CFCs, EryP-colony-forming cells; CFU-M, colony-forming unit-macrophage; CFU-Mix, CFU-EryP and macrophage mixed.

To investigate what kind of cells were differentiated from ciPSCs at various time points in early differentiation stage, we performed the flow cytometric analysis. Almost all ciPSCs expressed a pluripotency marker, SSEA-1, on day 0, whereas they were immediately differentiated with the loss of the SSEA-1 expression by day 3 (Figure 2A). Instead, the CD34^+^ hemangioblast/HE-like cells were enriched from days 3 to 5 (Figure 2B). Even after day 5, CD34^+^/CD45^−^ hemangioblast/HE-like cells were detected but their rates gradually decreased from day 6 (Figure 2C). The CD45^+^ hematopoietic cells were detected from days 6 to 9, while the CD34^+^/45^+^ HSPCs were mainly observed on days 6 and 7 and immediately disappeared by day 9 (Figure 2C). As the HSPCs were likely to be included in the differentiated cell population on day 7, we performed the hematopoietic colony-forming assay to clarify their hematopoietic differentiation potentials. They gave rise to a lot of small red colonies which seemed to be the primitive erythroid progenitors known as EryP-colony-forming cells (CFCs),^35^^,^^36^ a few colony-forming units-macrophage (CFU-M), and EryP-CFCs and macrophages mixed colonies (Figure 2D).

### Gene expression analysis of the differentiated cells in RBC differentiation process

**Figure 3.**
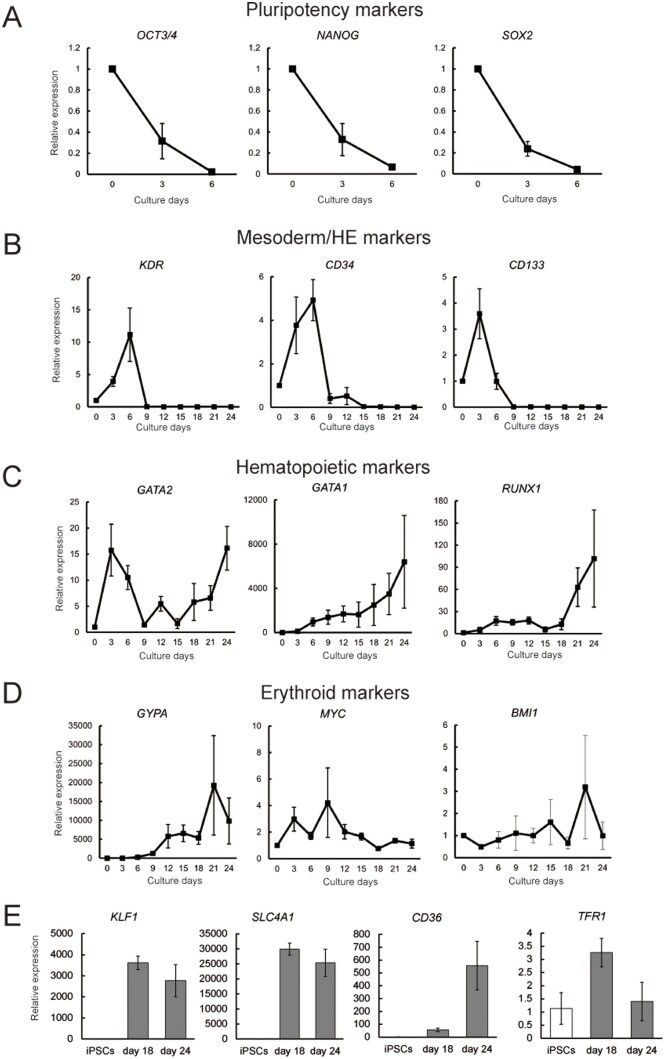
qRT-PCR analysis of differentiated cells. (A) qRT-PCR of differentiated cells for pluripotency markers. *β-ACTIN* was used as the housekeeping gene. Relative gene expression of the differentiated cells at each time point. Data are shown as the mean ± SD (*n* = 3). (B) qRT-PCR of differentiated cells for mesoderm/HE markers. *β-ACTIN* was used as the housekeeping gene. Relative gene expression of the differentiated cells at each time point. Data are shown as the mean ± SD (*n* = 3). (C) qRT-PCR of differentiated cells for hematopoietic markers. *β-ACTIN* was used as the housekeeping gene. Relative gene expression of the differentiated cells at each time point. Data are shown as the mean ± SD (*n* = 3). (D) qRT-PCR of differentiated cells for erythroid markers. *β-ACTIN* was used as the housekeeping gene. Relative gene expression of the differentiated cells at each time point. Data are shown as the mean ± SD (*n* = 3). (E) qRT-PCR of differentiated cells on days 18 and 24 for additional erythroid markers. HE: hemogenic endothelium. *β-ACTIN* was used as the housekeeping gene. Relative gene expression of the differentiated cells at each time point. Data are shown as the mean ± SD (*n* = 3).

The expression levels of the pluripotency markers *OCT3/4*, *NANOG*, and *SOX2* in differentiated cells decreased from days 0 to 6 (Figure 3A). In contrast, the expression levels of the mesoderm marker *KDR* and the hematopoietic endothelium and progenitor cell markers *CD34* and *CD133* peaked at day 3 or 6 and then rapidly declined by day 9 (Figure 3B). The expression level of *GATA2*, one of the important genes for hematopoiesis, increased at day 3 and after day 15 (Figure 3C). The expression levels of the hematopoietic progenitor markers *GATA1* and *RUNX1* increased as differentiation progressed (Figure 3C). Furthermore, the erythroid marker *GYPA* started to express around day 9 and kept high expression through the differentiation process. We also investigated the expression levels of other erythroid markers, *MYC* and *BMI1*, which play a critical role for the stemness of hematopoietic and erythroid progenitor cells and megakaryocytes.^37^^,^^38^ Interestingly, *MYC* expression was elevated from days 0 to 15 (2- to 4-fold compared to day 0) and declined to baseline levels by day 18 (Figure 3D). In contrast, *BMI1* expression remained generally low throughout differentiation, with a transient increase at day 21 that showed large variability among replicates (Figure 3D).

Following the induction of erythroid lineage commitment, we further examined the expression of additional erythroid-associated genes at later stages of differentiation (days 18 and 24). The erythroid transcription factor *KLF1*, a key regulator of erythroid maturation, and the erythrocyte membrane protein *SLC4A1* (Band 3) showed robust upregulation by day 18 and remained highly expressed at day 24 (Figure 3E). Expression of the early erythroid marker *CD36* was relatively low at day 18 but markedly increased by day 24 (Figure 3E). The transferrin receptor *TFR1* (CD71), which is associated with active erythropoiesis and iron uptake, was elevated at day 18 and decreased slightly by day 24 while remaining above baseline levels (Figure 3E). Collectively, these results further support the progressive acquisition of erythroid characteristics during differentiation and confirm the induction of late-stage erythroid gene expression in the differentiated cells.

### Hemoglobin expression in ciPSC-derived RBCs

**Figure 4.**
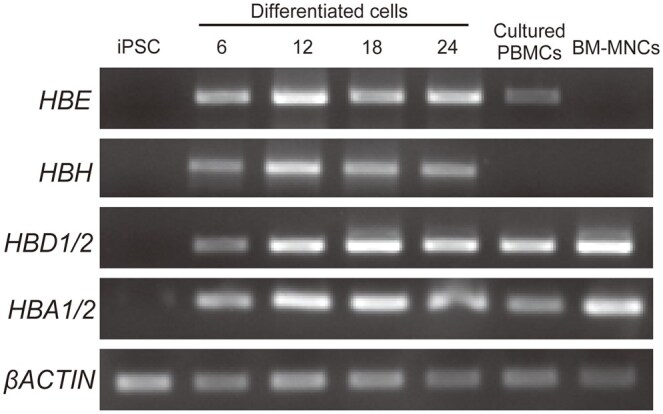
Hemoglobin gene expression analysis of differentiated cells. RT-PCR of the differentiated cells for *HBE*, *HBH*, *HBD1/2*, *HBA1/2*. Cultured PBMCs and BM-MNCs were used as positive controls. *β-ACTIN* was used as the housekeeping gene. *HBD1/2: HBD1* and *HBD2*, *HBA1/2: HBA1* and *HBA2*. The primers designed for *HBD1/2* and *HBA1/2* cross-amplify both genes due to the high similarity of their sequences. PBMCs: peripheral blood mononuclear cells, BM-MNCs: bone marrow mononuclear cells.

It is reported that hPSC-derived RBCs predominantly expressed the embryonic/fetal type but not adult type of hemoglobin.^18^^,^^20^^,^^21^^,^^39–41^ We investigated which types of hemoglobin ciPSC-derived RBCs express. In canines, the β-globin cluster has 5 β-globin genes: 2 embryonic/fetal-like genes (*HBE* and *HBH*) and 3 adult-like genes (*HBD1*, *HBD2*, and *HBB*). HBD1 and HBD2, which have the same protein sequence, are the primary adult β-globin instead of *HBB* in humans.^42^ Therefore, we performed reverse transcription PCR for each canine β-globin gene and adult-like α-globin genes, *HBA1* and *HBA2* using ciPSC-derived cells at some time points and adult cells isolated from adult dogs. In the cultured PBMCs and bone marrow mononuclear cells, *HBA1*, *HBA2*, *HBD1*, and *HBD2* were mainly detected (Figure 4). In contrast, expression of all examined hemoglobin genes was detected in the differentiated cells at each time point (Figure 4).

### GYPA expression in ciPSC-derived RBCs by generation of GYPA-EGFP reporter ciPSC lines

**Figure 5.**
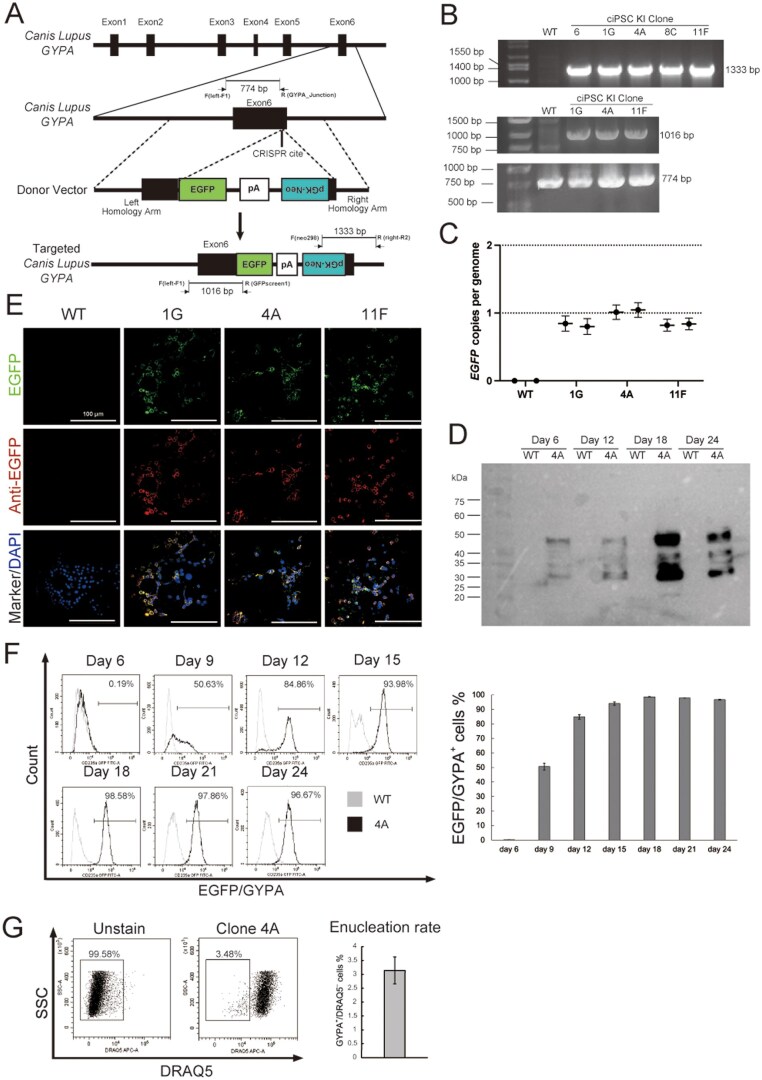
Generation of GYPA-EGFP reporter ciPSC lines and analysis of GYPA expression in differentiated cells. (A) CRISPR-Cas9–mediated genome editing of the GYPA locus. A fragment of EGFP cDNA was introduced in-frame to the sixth exon of the wild-type GYPA gene using donor vectors harboring EGFP, poly A (pA), and pGK-Neo selection cassette. Horizontal arrows show the positions of the forward (F) and reverse (R) primers used in panels (B). (B) PCR using genome DNAs isolated from each cell clone. Top panel: right junction PCR. Center panel: left junction PCR. Bottom panel: PCR using primers spanning the CRISPR-Cas9 cut site. WT, wild type; knock-in clones, 1G, 4A, and 11F. (C) Digital PCR analysis of *EGFP* copy number in knock-in iPSC clones. *MC1R* was used as a single-copy reference gene. Each point represents an individual technical replicate and error bars indicate the 95% CI reported for each measurement. WT, wild type; knock-in clones, 1G, 4A, and 11F. (D) Western blot for EGFP using differentiated cells derived from clone 4A on days 6, 12, 18, and 24. Multiple bands were revealed, which reflect differently glycosylated forms of this heavily O-glycosylated sialoglycoprotein. (E) Immunocytochemistry of differentiated cells derived from each ciPSC clone for EGFP. Top panels show EGFP expression by themselves without antibody. Center panels show EGFP expression detected by anti-GFP antibody. Bottom panels show the merge of the other two images and DAPI. WT, wild type, day 18; 1G, clone 1G, day 18; 4A, clone 4A, day 24; 11F, clone 11F, day 24. Scale bar = 100 µm. (F) Flow cytometric analysis of differentiated cells derived from clone 4A for EGFP at each time point. Upper panels show the histograms at each time point. Gray line: wild type (WT). Black line: differentiated cells derived from clone 4A (4A). Lower panel show the percentages of EGFP^+^ cells at each time point. Data are shown as the mean ± SD (*n* = 3). (G) Quantification of enucleation in differentiated erythroid cells. Day 24 differentiated cells derived from GYPA-EGFP reporter (clone 4A) were stained with the DNA dye DR and analyzed by flow cytometry. Cells were gated on GYPA^+^ erythroid cells (EGFP^+^ cells in the reporter line), and the percentage of DR^−^ cells within this population was quantified as enucleated erythrocytes. Data are shown as the mean ± SD (*n* = 3).

GYPA is a well-known marker specific to RBCs and is also considered a primitive hematopoietic cell marker.^22^ It could serve as a valuable indicator for both quantifying differentiation efficiency and elucidating the hematopoietic program in our RBC differentiation protocol using ciPSCs. However, some previous reports suggested that anti-human antibodies against GYPA are unlikely to work for canine cells.^24^^,^^43^ Our flow cytometric analysis for canine RBCs isolated from the blood samples of 3 healthy adult beagles using the same antibody as the previous report^43^ showed that no GYPA^+^ cells were detected in all dogs (data not shown). To monitor GYPA expression in ciPSC-derived cells without antibodies, we next tried to establish the GYPA-EGFP reporter ciPSC lines. In this experiment, we used the OPUiD05-A line which could be the most efficiently differentiated into RBCs. A fragment of the EGFP cDNA, poly A, and drug selection cassettes were introduced into canine GYPA allele by CRISPR-Cas9–mediated genome editing (Figure 5A). After G418 selection, successful homologous recombination was confirmed in at least 3 independent clones (1G, 4A, and 11F) by PCR analysis of both the right and left junctions using genomic DNA, followed by Sanger sequencing (Figure 5B). Polymerase chain reaction analysis using primers spanning the CRISPR-Cas9 cut site showed that all clones retained a WT-derived allele, suggesting mono-allelic integration of knock-in cassette (Figure 5B). In addition, quantitative PCR analysis of genomic DNA showed no amplification of the ampicillin resistance or Ori sequences in any of the clones, indicating the absence of random integration of vector-derived sequences into the host genome (data not shown). Digital PCR analysis normalized to the single-copy reference gene *MC1R* demonstrated that all 3 knock-in clones carried approximately 1 *EGFP* copy per genome, whereas no *EGFP* copies were detected in the wild-type control (Figure 5C). These results are consistent with mono-allelic integration of the EGFP cassette in each clone without the presence of additional random integrations. All clones were confirmed to keep the ciPSC characteristics such as normal karyotypes, the pluripotency markers expression, and the differentiation capacities *in vitro* and *in vivo* even after genome editing (Figure S1A-E, see online supplementary material for a color version of this figure). All clones were differentiated into RBCs with our protocol. The differentiated cells at any differentiation time point derived from all 3 clones were confirmed to express EGFP via western blotting (Figure 5D). In addition, immunostaining showed that the cell surface of most differentiated cells on days 18 and 24 expressed EGFP, and they co-localized with anti-EGFP antibody staining (Figure 5E). Finally, flow cytometric analysis indicated that ciPSCs-derived GYPA^+^ RBCs started to emerge between days 6 and 9 and then immediately increased the positive rates until day 18, reaching higher than 96% (Figure 5F). To quantitatively evaluate erythroid maturation, we assessed enucleation at day 24 of differentiation. Flow cytometric analysis revealed that approximately 3% of GYPA^+^ cells were DR-negative, indicating that a subset of differentiated erythroid cells underwent enucleation under the current culture conditions (Figure 5G).

### RBC differentiation of multiple ciPSC lines

**Figure 6.**
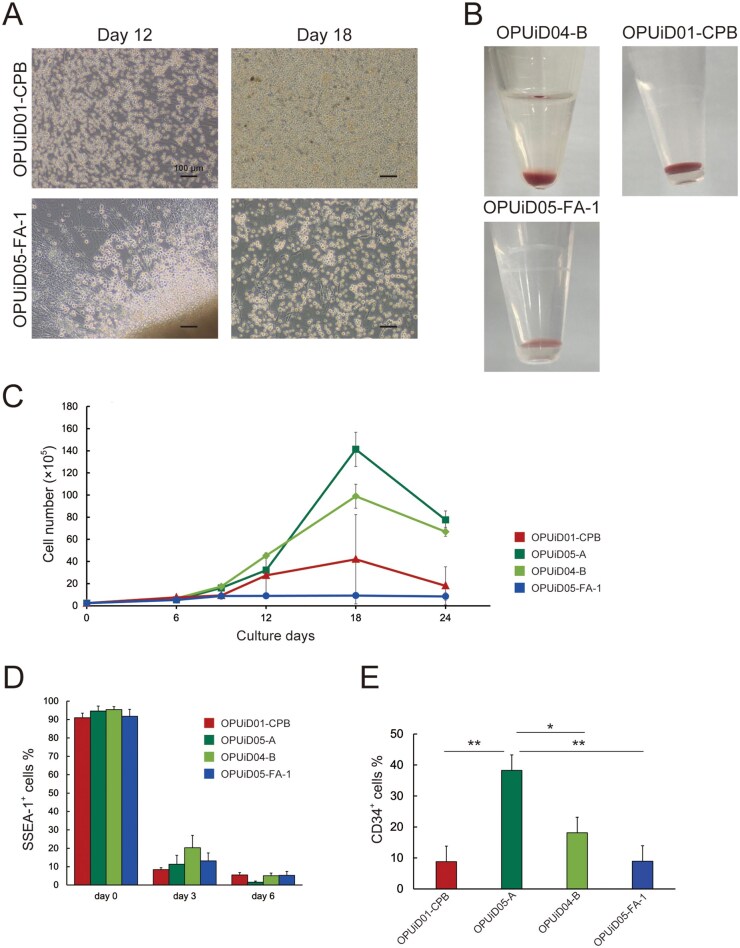
RBC differentiation using multiple ciPSC lines. (A) Morphology of differentiated cells on days 12 and 18. Upper panels showed the images of the cells derived from OPUiD01-CPB. Lower panels showed the images of the cells derived from OPUiD05-FA-1. Scale bar = 100 µm. (B) Morphology of differentiated cell pellets on day 24. (C) Cell number of differentiated cells derived from each cell line at each time point. Data are shown as the mean ± SD (*n* = 3). (D) Flow cytometric analysis of differentiated cells derived from each ciPSC line for SSEA-1 on days 0, 3, and 6. Data are shown as the mean ± SD (*n* = 3). (E) Flow cytometric analysis of differentiated cells derived from each ciPSC line for CD34 on day 6. Data are shown as the mean ± SD (*n* = 3). *P < .05, **P < .01.

Having confirmed that 1 ciPSC line can be differentiated into RBCs, we next proceeded to evaluate whether our protocol could be applicable for 3 ciPSC lines: 2 already established ciPSC lines, PBMC-derived line (OPUiD04-B) and DF-derived line (OPUiD05-FA-1), and 1 new line, cultured PBMC-derived line (OPUiD01-CPB), which was generated in this study. The PBMCs were isolated from the healthy dogs and PBMCs were cultured in Stemline II-based medium supplemented with EPO, SCF, and IGF1 for 7 days, resulted in the expansion of erythroid progenitor–like cells (Figure S2A, see online supplementary material for a color version of this figure). The cultured PBMCs were reprogrammed using the new type of SeV encoding canine 6 factors, which we recently reported,^30^ with the modification that can be automatically erased by expressing microRNA-302 expressed in PSCs.^44^ We confirmed that established ciPSC lines had all ciPSC characteristics such as the colony morphologies, SeV removal, normal karyotypes, pluripotency markers expression, and differentiation capacities both *in vitro* and *in vivo* (Figure S2B-G, see online supplementary material for a color version of this figure). We next differentiated the 3 ciPSC lines into RBCs with the same protocol the experiment before and compared the results to that of OPUiD05-A line. PBMC-derived OPUiD04-B and cultured PBMC–derived OPUiD01-CPB could be differentiated with the similar morphology expression to OPUiD05-A (Figure 6A and B). In contrast, DF-derived OPUiD05-FA-1 likely differentiated into neural-like cells with neurite and a few spherical hematopoietic cells with low levels of hemoglobinization (Figure 6A and B). Compared the cell numbers among ciPSC lines, PBMC-derived lines could have higher numbers of differentiated cells than other lines (Figure 6C). The most efficient cell line, OPUiD05-A, generated an average of 32.3 RBCs per ciPSC following 24 days of differentiation. The SSEA-1^+^ cells decreased in 6 days after differentiation in all ciPSC lines, whereas the rate of CD34^+^ cells on day 6 was significantly higher in OPUiD05-A line than that in other ciPSC lines (Figure 6D and E).

## Discussion

RBC transfusion is now essential in saving lives in various circumstances. Many researchers worldwide are working on large-scale RBC production technologies, as PSCs could provide an unlimited source of RBCs for transfusion. However, few studies aim to develop appropriate preclinical models to translate these advanced technologies into human medicine. Canine-induced pluripotent stem cell–derived RBC transfusion into dogs could be an excellent model for evaluating the safety and therapeutic efficacy of this treatment. However, RBC differentiation using ciPSCs has not yet been achieved. Here, we developed an RBC differentiation protocol using ciPSCs by combining and modifying 2 hematopoietic cell differentiation protocols for hPSCs.^33^^,^^34^ Our protocol demonstrated that ciPSC-derived EBs could stably differentiate into hemoglobinized RBCs, progressing through polychromatic and orthochromatic erythroblast–like cells. This finding indicates a preliminary approach, providing an early basis for further optimization and evaluation toward future transfusion-related applications.

Importantly, our protocol did not require cocultures with any animal-derived feeder cells and utilized a chemically defined medium for the early stage until day 12. In several hPSC studies, coculturing with feeder cells derived from mice, such as 10T1/2 and OP9 cell lines, was necessary for efficient differentiation into hematopoietic cells/RBCs.^18^^,^^19^ Although one study reported platelet generation from ciPSCs using OP9 cell lines,^45^ undefined components can affect reproducibility due to batch-to-batch variability in serum and cell lines, complicating clinical applications. Therefore, our protocol’s lack of undefined components makes it easier to optimize or modify for further clinical and research applications.

In the early differentiation stage, endothelial-like cells expanded with increased expression of *KDR*, *CD133*, and *CD34*, markers of hemangioblast/HE or endothelial progenitor cells in humans.^46–48^ This was followed by the emergence of spherical CD45^+^ hematopoietic cells from endothelial-like cells, with some important genes for the hematopoiesis program upregulated. These data suggest that our protocol could recapitulate the early stage of mammalian hematopoiesis, including HE differentiation and hematopoietic cell emergence through endothelial-to-hematopoietic transition, as observed in hPSC hematopoietic cell differentiation.^34^^,^^47^^,^^48^

In contrast, the CD34^+^/CD45^+^ HSPCs, which had only EryP-CFC and CFU-M colony formation capacities, were detected around day 7 and then became undetectable. Instead, *GYPA* gene expression began increasing between days 3 and 6, and hemoglobinized RBCs expressing all types of hemoglobin genes, including embryonic and fetal types, increased. These data suggest that our protocol predominantly mimicked the primitive erythropoiesis program consistent with previous hPSC studies.^18^^,^^20^^,^^21^^,^^39–41^^,^^49^ To produce definitive RBCs for transfusion applications, it is essential to understand how to control primitive and definitive erythropoiesis. Future research should investigate addressing this important problem in RBC differentiation protocols using ciPSCs.

Reporter ciPSC lines generated by CRISPR-Cas9–mediated genome editing have only rarely been reported,^50^ whereas reporter hPSCs have been previously reported. Although there are several reports on ciPSC generation,^30^^,^^51–54^ genome editing of ciPSCs has remained limited, likely due to the lack of a stable culture method for ciPSCs, unlike hPSCs. We have reported high-quality ciPSC generation and feeder-free culture systems for ciPSCs.^29^^,^^55^ These enabled single-cell passages, essential for expanding genome-edited ciPSC clones from each single cell. Our strategy for ciPSC genome editing and the generation of reporter ciPSC lines would be a powerful tool for investigating optimal differentiation methods into various cell types and developing hereditary disease models.

The generation of GYPA-EGFP reporter ciPSC lines allowed us to visualize GYPA expression in iPSC-derived RBCs, establishing a system for evaluating the RBC differentiation efficiency. This system may facilitate investigations into key cytokines, essential signaling pathways, and the optimal timing for modulating these pathways during RBC differentiation from ciPSCs. Notably, previous hPSC research indicated that GYPA serves as a specific marker to distinguish primitive hematopoietic cells from definitive hematopoietic cells in the early stage of differentiation.^23^ Additionally, manipulating pathways—such as inhibiting Activin/Nodal signaling while activating Wnt/β-catenin signaling—could enhance the generation of definitive hematopoietic progenitors/RBCs, improving both their maturation capacity and adult hemoglobin expression.^23^ Although further testing is required to confirm whether GYPA is a specific marker for primitive hematopoietic cells in canines, as it is in humans, if proven, our reporter ciPSCs could greatly aid in the development of definitive hematopoietic cell/RBC differentiation protocols.

In this study, the GYPA-EGFP reporter lines contain a PGK-Neo selection cassette flanked by loxP sites, allowing for Cre-mediated excision. Because the cassette was not removed in the current experiments, we cannot exclude the possibility that retention of the PGK-Neo/loxP sequences may influence GYPA expression, its downstream function, or aspects of erythroid maturation. In future work, we plan to excise the cassette using Cre recombinase to eliminate any potential disruption of normal GYPA expression and function.

Cultured canine PBMCs seemed to include different populations, such as erythroblast-like cells. This outcome aligns with previous findings where human PBMCs were cultured in similar media.^56–58^ Furthermore, using the SeV vector incorporating 6 canine factors, we successfully generated footprint-free ciPSCs from cultured PBMCs. The cell lines exhibited characteristics of high-quality iPSCs, as confirmed by teratoma formation capacity. These findings suggest that the new SeV vector is a valuable tool for producing high-quality, footprint-free ciPSCs from blood cells, which are easily accessible in clinical settings, in addition to previously reported sources such as CEF, skin DFs, and urinary-derived cells.^30^

Interestingly, RBC differentiation efficiency varied among ciPSC lines. Notably, hematopoietic cells–derived ciPSC lines exhibited a tendency toward higher differentiation efficiency compared to DF-derived ciPSC lines. Previous studies have reported that hiPSC lines derived from blood cells have higher efficiency in differentiating into hematopoietic cells than those derived from DFs.^59^ Multiple pieces of evidence indicate that the type of somatic cells used to generate iPSCs can influence the efficiency of differentiation into specific lineages.^59–61^ Conversely, some studies argue that the differences in differentiation potential are more significantly affected by the donor or the iPSC lines themselves rather than the origin cell type of iPSCs.^62–64^ We have previously observed variability in the expression of pluripotency- and lineage-associated markers among ciPSC lines, which may reflect differences arising from the reprogramming process, donor-related factors, or extended culture.^30^^,^^57^ These factors may contribute to the divergent differentiation tendencies seen in this study; however, the underlying mechanisms remain unclear. Further investigations will be required to clarify the causes of line-to-line variability. In addition, the number of cell lines used in this study was limited; hence, future research should involve comparing the differentiation efficiencies of a larger number of ciPSC lines derived from various types of somatic cells. This will help identify ciPSC lines that are more suitable for hematopoietic/RBC differentiation.

Although the PBMC-derived ciPSC lines showed the highest RBC production among the lines tested in this study, the overall yields and enucleation rates remain insufficient for any translational or large-scale application. To overcome these limitations, previous studies have proposed several strategies, including the replacement of animal-derived components and growth factors with synthetic or chemically defined alternatives to improve differentiation efficiency, scalability, and cost-efficiency.^65^^,^^66^ Moving forward, further optimization of the differentiation protocol will be essential to enhance yield, reproducibility, cost-effectiveness, and enucleation efficiency, thereby supporting future progress toward practical application.

Despite the progress achieved in this study, several limitations should be acknowledged. First, we did not assess key functional properties of the ciPSC-derived RBCs, including oxygen-carrying capacity, deformability, and *in vivo* survival, which are essential for determining whether these cells can perform as mature, transfusion-relevant RBCs. Furthermore, although hemoglobin gene expression was detected in the ciPSC-derived RBCs, we were unable to confirm hemoglobin composition at the protein level. Future work incorporating functional assays, quantitative assessments of hemoglobin isoforms, and strategies to enhance differentiation efficiency will be necessary to advance the clinical and translational potential of this approach.

## Conclusion

We successfully established a protocol for differentiating ciPSCs into RBCs. This method demonstrated that ciPSCs could differentiate into hemoglobinized RBCs, progressing through stages that include the formation of HSPCs and polychromatic and orthochromatic erythroblast–like cells. Furthermore, we generated GYPA-EGFP reporter ciPSCs using CRISPR-Cas9–mediated genome editing. These reporter ciPSCs enabled us to visualize GYPA expression in ciPSC-derived RBCs, confirming that GYPA could be a good surface marker for ciPSC-derived RBCs, similar to human erythrocytes and that our protocol could produce more than 95% GYPA^+^ RBCs. This study opens new possibilities for translational research, offering a promising approach for developing safe and effective transfusion therapies that bridge veterinary and human medicine. By leveraging the unique advantages of ciPSCs, we can further explore and optimize RBC production, potentially addressing the global challenges of blood supply shortages and enhancing the scope of regenerative medicine.

## Supplementary Material

szag058_Supplementary_Data

## Data Availability

The data underlying this article will be shared on reasonable request to the corresponding author.
