# Supplementary material for: Red blood cell differentiation using canine-induced pluripotent stem cells
Source: Stem Cells Transl Med. 2026 Jul 20;15(8):szag058. doi: 10.1093/stcltm/szag058 (PMC13381058; doi:10.1093/stcltm/szag058)
Supplement: szag058_Supplementary_Data [file szag058_supplementary_data.pdf]

Supplementary Materials

Red blood cell differentiation using canine induced pluripotent stem cells

Kazuto Kimura[1, 4], Masaya Tsukamoto[1], Kohei Shishida[1], Hiroko Sugisaki[1], Jun Katahira[2], Miyuu Tanaka[3], Mitsuru Kuwamura[3], Amir Kol[4], Mika Okada[5], Minoru Iijima[5], Mahito Nakanishi[5], Kikuya Sugiura[1], Shingo Hatoya[1, *]

[1]Department of Advanced Pathobiology, Laboratory of Cell Pathobiology, Graduate School of Veterinary Science, Osaka Metropolitan University, Izumisano, Osaka 598-8531, Japan

[2] Department of Integrated Functional Biosciences, Laboratory of Cellular Molecular Biology, Graduate School of Veterinary Science, Osaka Metropolitan University, Izumisano, Osaka 598-8531, Japan

[3] Department of Integrated Structural Biosciences, Laboratory of Veterinary Pathology, Graduate School of Veterinary Science, Osaka Metropolitan University, Izumisano, Osaka 598-8531, Japan

[4] Department of Pathology, Microbiology and Immunology, School of Veterinary Medicine, University of California, Davis, Davis, CA 95616, USA.

[5] TOKIWA-Bio Inc., Tsukuba, Ibaraki 305-0047, Japan

[*] **Correspondence information**

Shingo Hatoya, DVM, Ph.D., Osaka Metropolitan University, Izumisano, Osaka 598-8531, Japan; Phone & Fax: +81-72-463-5379; Email: hatoya@omu.ac.jp

Figure S1. Characterization of genome edited ciPSC clones. (A) Karyotype analysis of each clone. clone 1G; passage 26, clone 4A; passage 26, clone 11F; passage 26. (B) qRT-PCR of each ciPSC clone for *OCT3/4*, *NANOG*, and *SOX2*. *β-ACTIN* was used as the housekeeping gene. Relative gene expression to Wild type (WT). Data are shown as the mean ± standard deviation (n = 3). $^{*}p < 0.05$, $^{**}p < 0.01$. (C) Immunocytochemistry of each clone for pluripotent markers OCT3/4, NANOG, and SOX2. Scale bar = 100 µm. (D) Immunocytochemistry for differentiation markers of each ciPSC clone after spontaneous differentiation. Ectodermal marker TUBB3 and PAX6, mesodermal marker α-SMA, and endodermal marker SOX17 and FOXA2. Scale bar = 100 µm. (G) Teratoma formation of each ciPSC clone. The image on the upper left in each ciPSC clone shows the testis with a tumor (1G; left, 4A; left, 11F; right) and normal testis (1G; right, 4A; right, 11F; left). Teratomas contain the three germ layers: ectoderm; squamous epithelium and hair follicles or neural tissues, mesoderm; cartilage, and endoderm; respiratory epithelium-like cells. Scale bar = 1 cm (teratoma image) or 100 µm (histological images).

Figure S2. Generation of ciPSCs from cultured PBMCs. (A) Morphology of the cells before (left) and after (right) culture. Scale bar = 100 µm. (B) Morphology of ciPSCs. OPUiD01-CPB, passage 13. (C) RT-PCR of ciPSCs for *SeV*. CEFs and infected CEFs as negative and positive controls, respectively. CEF; canine embryonic fibroblast. Infected CEF; SeV infected CEF. *β-ACTIN* was used as the housekeeping gene. (D) Karyotype analysis of OPUiC01-UF-A at passage 14. (E) Immunocytochemistry of OPUiD01-CPB at passage 25 for pluripotent markers OCT3/4, NANOG, and SOX2. Scale bar = 100 µm. (F) Immunocytochemistry for differentiation markers of OPUiD01-CPB after spontaneous differentiation. Ectodermal marker TUBB3, mesodermal marker DESMIN, and endodermal marker SOX17. Scale bar = 100 µm. (G) Teratoma formation of OPUiD01-CPB. The image on the upper left shows the testis with a tumor (right) and normal testis (left). Teratomas contain the three germ layers: ectoderm; neural tissues, mesoderm; connective tissues, and endoderm; respiratory epithelium-like cells. Scale bar = 1 cm (teratoma image) or 100 µm (histological images).

Table S1 Primers for RT-PCR and qRT-PCR

Sequence of each primer

Gene Name		Sequence (5'→3')
KDR	Forward	GACCAAGGGTGGTACACCTG
	Reverse	GGCTTCCACCGAAGATTCCA
CD34	Forward	GGACCACAGCAACCATCTCA
	Reverse	ACCATTTCCAGGTAGCAGGC
CD133	Forward	GGAAGGCGAGGAGCACTATC
	Reverse	TGGAGGAAGTCCTGAGCAGA
GATA1	Forward	AACAAGCCCAGGTCATACCC
	Reverse	CCGGTGGTGACGATACCAAG
GATA2	Forward	CAGAACCGGCCACTCATCAA
	Reverse	AATTTGCACAACAGGTGCCG
RUNX1	Forward	TTTGTCGGTCGGAGTGGAAG
	Reverse	TCTGCCTTGTGTTTCGAGGT
MYC	Forward	CAGCGACTCGGAGGAAGAA
	Reverse	CGATTCGGACCTTTTGGCAG
BMI1	Forward	AGAAACCTAAGGAGGAGGTGAATG
	Reverse	AAAGGTTCCTCTTCGTACATGACA
GYPA	Forward	CCGACACAAGCAGGTTTCATT
	Reverse	CTTGCTTTTTGTCCCCCATCTG
KLF1	Forward	GACTTTCTCAAGTGGTGGCG
	Reverse	CTCTCGTCGTCTTCGTCCTC
CD36	Forward	GGTCCTTACACATACAGAGTTCGT
	Reverse	AGGTTCAAAGATGGCACCGT
TFR1	Forward	GGGACAGAGAGAACAGAACCC
	Reverse	TTCATTCAGCCACCTCATGGT
SLC4A1	Forward	GCTGTGCTGTTTGGCATCTT
	Reverse	CCAGGTCTTTACCCGCTTGA
HBD	Forward	GTGGACGAAGTTGGCGGTGAG
	Reverse	CTTTGCCAAAGTGGTGAGCCAG
HBH	Forward	TCACCAGCAAGTTCCCAGACACC
	Reverse	CTCTGGCAATACCGACATAATTGGAC

HBE	Forward	CTGTAAGCTCTCAGACCTGACATC
	Reverse	GTACTGCTGAACTAGAAAGAGGACTC
HBA	Forward	ACTTCCCGCACTTCGACCTGAG
	Reverse	CTTGAAGTTGACGGGGTCCACG
β-ACTIN	Forward	CAGCAAATGTGGATCAGC
	Reverse	CCAATCTCATCTCGGTTTC
SeV	Forward	GGAAGGAATCGGCTCAGTGATG
	Reverse	GGGCCGTGTTCATGGTCAC
OCT3/4	Forward	CTGAAGCAGAAGAGGATCAC
	Reverse	GCCGCAGCTTACACATATTC
SOX2	Forward	AACCCCAAGATGCACAACTC
	Reverse	CGGGGCCGGTATTTATAATC
NANOG	Forward	CTAGGGACCCTTCTCCAATG
	Reverse	CTTCTGTTTCTTGCCCTGG
AmpR	Forward	GGCTGGCTGGTTTATTGCTG
	Reverse	AACTACGATACGGGAGGGCT
Ori	Forward	CGCCTACATACCTCGCTCTG
	Reverse	ATCGTCTTGAGTCCAACCCG

Table S2 Antibodies for Immunocytochemistry and Flowcytometry

Primary antibodies for immunocytochemistry

Antigen	monoclonal/polyclonal	Host	Clone	Cat. No.	Source	Final concentration
human OCT3/4	monoclonal	mouse IgG2b	C-10	sc-5279	Santa Cruz Biotechnology	2 μg/ml
human NANOG	polyclonal	rabbit IgG	-	500-P236	Peprotech	2 μg/ml
human SOX2	monoclonal	mouse IgG1	E-4	sc-365823	Santa Cruz Biotechnology	2 μg/ml
human TUBB3	monoclonal	mouse IgG1	TU-20	MAB1637	Sigma-Aldrich	1 μg/ml
mouse Pax6	polyclonal	rabbit IgG	-	910301	Biolegend	2 μg/ml
mouse α-SMA	monoclonal	mouse IgG2a kappa	1A4	M0851	Agilent	1:500
mouse Desmin	polyclonal	rabbit IgG	-	ab82506	abcam	1 μg/mL
human FOXA2	polyclonal	rabbit IgG	-	720061	Thermo Fisher Scientific	2.5 μg/mL
human SOX17	polyclonal	goat IgG	-	AF1924	R&D systems	2 μg/ml

Secondary antibodies for immunocytochemistry

Antigen	Cat.no.	Source	Final concentration
Goat anti-Mouse IgG, Alexa Fluor 488	A11029	Thermo Fisher Scientific	1 μg/ml
Goat anti-Rabbit IgG, Alexa Fluor 546	A11010	Thermo Fisher Scientific	4 μg/ml
Rabbit anti-Goat IgG, Alexa Fluor 488	A27012	Thermo Fisher Scientific	1 μg/mL

Primary antibody for Flowcytometry

Antigen	monoclonal/polyclonal	Host	Clone	Cat. No.	Source	Final concentration
normal mouse IgM	-	-	-	sc-3881	Santa Cruz Biotechnology	40 μg/ml
Mouse IgG1 kappa Isotype Control, PE	-	-	-	12-4714-42	Thermo Fisher Scientific	2.5 μg/mL
Rat IgG2b kappa Isotype Control, FITC	-	-	-	11-4031-82	Thermo Fisher Scientific	2.5 μg/mL
mouse SSEA-1	monoclonal	mouse IgM	480	sc-21702	Santa Cruz Biotechnology	2 μg/ml
Canine CD34, PE	monoclonal	mouse IgG1 kappa	1H6	12-0340-42	Thermo Fisher Scientific	2.5 μg/mL
Canine CD45, FITC	monoclonal	Rat IgG2b kappa	YKIX716.13	11-5450-42	Thermo Fisher Scientific	2.5 μg/mL

-

Secondary antibody for Flowcytometry

Antigen	Cat.no.	Source	Final concentration
Goat anti-Mouse IgM, Cy3	AP128C	Sigma Aldrich	1 μg/ml

Table S3 Sequence of sgRNA and primers for screening PCR

sgRNA

	Sequence (5'→3')
sense	CACCGTTGTTTCTACAGAACCTCA
antisense	AAACTGAGGTTCTGTAGAAACAAC

primers for screening

primer	Sequence (5'→3')
right-R2 (F)	ATCACTTGGGCTTATGCTTCAGGTGACATC
neo298 (R)	AGGGGAGGAGTAGAAGGTGGCGCGAAGGGG
Left-F1 (F)	CAACTTCCATTGGGTGACTTCTATCATGATCC
EGFP-ScreenA1 (R)	ATGGCGGACTTGAAGAAGTCGTGCTGCTTC
GYPA_junction (R)	ATTAGCACCAACCTGGATTTCTTGTTTCTACA

Table S4 Sequence of primers and probes for dPCR

Name	Sequence (5'→3')
K9-MC1R-F	GAAAGACTCTCCAAGAGGTAGTG
K9-MC1R-R	CCAGTCACATGGGTATCAATCA
K9-MC1R-Pr	5' HEX/ZEN/3' IBFQ: CTATGTTCCTGGTGAGGCTGCAGG
EGFP-dPCR-F	GCACAAGCTGGAGTACAACTA
EGFP-dPCR-R	TGTTGTGGCGGATCTTGAA
EGFP-dPCR-Pr	5' 6-FAM/ZEN/3' IBFQ: AGCAGAAGAACGGCATCAAGGTGA

Supplemental materials and methods

Culture medium composition

The feeder medium (FM) was composed of high-glucose Dulbecco's modified Eagle medium (DMEM; Nacalai Tesque) containing 10% FBS (Kibbutz Beik Haemek, Israel), 2 mM L-glutamine (Nacalai Tesque), 100 U/mL penicillin, and 100 µg/mL streptomycin (Nacalai Tesque). N2B27 medium consisted of DMEM/Nutrient Mixture F-12 Ham (Nacalai Tesque) supplemented with N2 supplement (1×, Thermo Fisher Scientific), B27 supplement (1×, Thermo Fisher Scientific), GlutaMAX (1×, Thermo Fisher Scientific), 0.1 mM minimal essential medium non-essential amino acids (MEM NEAA; Thermo Fisher Scientific), and 0.1 mM 2-mercaptoethanol (Sigma-Aldrich). Six small molecule compounds cocktail (6SMs) contained 10 µM Y-27632 (Nacalai Tesque), 0.5 µM PD0325901 (Fujifilm Wako Pure Chemical Corporation, Osaka, Japan), 3 µM CHIR99021 (Nacalai Tesque), 0.5 µM A83-01 (Nacalai Tesque), 10 µM Forskolin (Nacalai Tesque), and 50 µg/mL L-ascorbic acid (Sigma-Aldrich). The EB medium consisted of 10% FBS (Sigma-Aldrich), 10% KSR (Thermo Fisher Scientific), 1 mM sodium pyruvate (Nacalai Tesque), 2 mM L-glutamine, 100 U/mL penicillin, 100 µg/mL streptomycin, 0.1 mM MEM NEAA, and 0.1 mM 2-mercaptoethanol.

Preparation and culture of feeder cells and cultured canine PBMCs

MEFs were isolated from the fetuses of ICR mice (Japan SLC, Shizuoka, Japan). The head, visceral tissues, and liver were removed from the fetus. The remaining tissues were cut into small pieces and cultured in FM. MEFs were cultured in the FM in a 37 °C humidified incubator supplemented with 5% CO_2. MEFs were passaged by dissociation using 0.25% trypsin-EDTA (Nacalai Tesque) and treated with 10 µg/mL mitomycin C (Kyowa Kirin, Tokyo, Japan) for 2.5 h at passage 3. Inactivated MEFs were collected by 0.25% trypsin-EDTA and stored at −80 °C using BAMBANKER® (Nippon Zenyaku Kogyo). The peripheral blood

mononuclear cells (PBMCs) were obtained from beagle dogs as previously reporter.[1] PBMCs were used for reprogramming after 7d culture with the Stemline II based medium supplemented with 50 ng/ml canine stem cell factor (cSCF; R&D system), 3U/ml canine Erythropoietin (cEPO; R&D systems), and 20 ng/ml human insulin-like growth factor 1 (hIGF1; R&D systems).

Reprogramming of cultured PBMCs into ciPSCs using SeV

We recently reported a new type of Sendai virus vector (SeV) encoding canine 6 pluripotency-associated genes, Kruppel-like factor (KLF) 4, octamer-binding transcription factor (OCT) 3/4, sex-determining region Y-box (SOX) 2, C-MYC, NANOG, and Lin28A.[2] In this study, we used the modified SeV, which can be automatically erased by expressing microRNA-302 (miR-302) expressed in PSCs. Cultured PBMCs were reprogrammed using our previously reported protocol[1] with minor modification. In brief, cultured PBMCs were infected with the SeV at a multiplicity of infection (MOI) of 3. Infected cells were incubated at 37°C in 5% CO_2 for 16 h. The infected cells were resuspended in the Stemline II based medium and seeded at the density of 3×10^5 cells/well in MEF-seeded 12-well tissue culture plate. On day 1 post-infection, the medium was replaced with reprogramming media, N2B27 supplemented with 10 ng/mL human basic fibroblast growth factor (bFGF; Peprotech) and 6SMs. The media were replenished every day. On day 8 post-infection, the media were replaced with StemFit. Around day 14–21 post-infection, primary ciPSC-like colonies were mechanically picked and cultured onto iMatrix-511 silk-coated dish in StemFit to establish ciPSC lines.

Karyotyping analysis

The fiPSCs were incubated with 0.05 μg/mL colcemid (Thermo Fisher Scientific) for 1 h, trypsinized, and incubated with 0.075 M KCl at 37 °C for 20 min. The cells were fixed in acetic

acid: methanol (1:3), stained with quinacrine mustard and Hoechst 33258, and observed using confocal laser-scanning microscopy (LSM980; Carl Zeiss, Oberkochen, Germany).

in vitro differentiation assay

The *in vitro* differentiation ability of ciPSCs was evaluated as spontaneous differentiation via EB formation. fiPSCs were dissociated into single cells using TrypLE Select and then seeded in Nunclon™ Sphera™ 96-Well U-Shaped-Bottom Microplate (Thermo Fisher Scientific) at a density of 4.0×10^4 cells per well in the EB medium and were maintained for 12 days. The cells were analyzed by immunostaining.

Teratoma formation assay

Approximately 1×10^6 ciPSCs were injected into the testis capsule of NOD/SCID mice. The mice were euthanized by cervical dislocation after three months, and the tumors were fixed in 4% paraformaldehyde, paraffin-embedded, sectioned, and stained with hematoxylin and eosin.

Reference

1 Kimura K, Tsukamoto M, Tanaka M, et al. Efficient reprogramming of canine peripheral blood mononuclear cells into induced pluripotent stem cells. Stem Cells Dev 2021;30:79–90.

2 Tsukamoto M, Kimura K, Yoshida T, et al. Generation of canine induced pluripotent stem cells under feeder-free conditions using Sendai virus vector encoding six canine reprogramming factors. Stem Cell Reports 2024;19:141–157.
